# Strength of interference screw fixation of meniscus prosthesis matches native meniscus attachments

**DOI:** 10.1007/s00167-021-06772-9

**Published:** 2021-10-19

**Authors:** M. K. Bartolo, E. Provaggi, K. K. Athwal, S. Newman, M. A. Accardi, D. Dini, A. Williams, A. A. Amis

**Affiliations:** 1 Biomechanics Group, Mechanical Engineering Department, Imperial College London, London, SW7 2AZ UK; 2 Orthonika Ltd, London, UK; 3 Department of Surgery and Cancer, Imperial College London School of Medicine, London, UK; 4 Fortius Clinic, London, UK

**Keywords:** Knee, Meniscus, Fixation system, Meniscus replacement, Ovine, Pull-out strength, Slippage, Interference screws, Tensile, Creep

## Abstract

**Purpose:**

Meniscal surgery is one of the most common orthopaedic surgical interventions. Total meniscus replacements have been proposed as a solution for patients with irreparable meniscal injuries. Reliable fixation is crucial for the success and functionality of such implants. The aim of this study was to characterise an interference screw fixation system developed for a novel fibre-matrix-reinforced synthetic total meniscus replacement in an ovine cadaveric model.

**Methods:**

Textile straps were tested in tension to failure (*n* = 15) and in cyclic tension (70–220 N) for 1000 cycles (*n* = 5). The textile strap-interference screw fixation system was tested in 4.5 mm-diameter single anterior and double posterior tunnels in North of England Mule ovine tibias aged > 2 years using titanium alloy (Ti6Al4Va) and polyether-ether-ketone (PEEK) screws (*n* ≥ 5). Straps were preconditioned, dynamically loaded for 1000 cycles in tension (70–220 N), the fixation slippage under cyclic loading was measured, and then pulled to failure.

**Results:**

Strap stiffness was at least 12 times that recorded for human meniscal roots. Strap creep strain at the maximum load (220 N) was 0.005 following 1000 cycles. For all tunnels, pull-out failure resulted from textile strap slippage or bone fracture rather than strap rupture, which demonstrated that the textile strap was comparatively stronger than the interference screw fixation system. Pull-out load (anterior 544 ± 119 N; posterior 889 ± 157 N) was comparable to human meniscal root strength. Fixation slippage was within the acceptable range for anterior cruciate ligament graft reconstruction (anterior 1.9 ± 0.7 mm; posterior 1.9 ± 0.5 mm).

**Conclusion:**

These findings show that the textile attachment-interference screw fixation system provides reliable fixation for a novel ovine meniscus implant, supporting progression to in vivo testing. This research provides a baseline for future development of novel human meniscus replacements, in relation to attachment design and fixation methods. The data suggest that surgical techniques familiar from ligament reconstruction may be used for the fixation of clinical meniscal prostheses.

## Introduction

Meniscal injury significantly affects quality of life [32], and meniscal surgery is one of the most common orthopaedic surgical interventions worldwide [1, 7, 18, 32], with 1 million meniscal surgeries occurring annually in the United States alone [21]. Only approximately 15–35% of meniscus tears are repairable [8, 33]. When meniscus repair is not possible, the current standard of care is meniscectomy for symptomatic tears having failed non-surgical treatment. Although meniscectomy may alleviate symptoms in the short term, it increases the risk of the onset of osteoarthritis [7, 18, 21, 23, 27–29]. Other treatment options, including meniscal allograft transplants [22, 35] and partial replacement scaffolds [21, 23, 30, 34, 42], have limited success in long-term function and survivorship [14, 15, 41]. Novel total meniscus replacement devices have been proposed to fill this treatment gap [19, 26, 37].

Adequate fixation is crucial for the success and functionality of total meniscus replacements. Total meniscus replacements currently in development have used sutures for fixation in large animal models. Sutures connected to the anterior and posterior horns of the implant were passed through transosseous tunnels in the tibia and tied distally with a knot or endobutton™ [4, 16, 37, 38]. However, in vivo data reported implant extrusion and fixation ruptures with such fixation systems. One total meniscus prototype utilised a novel screw-type fixation method in transosseous tunnels for large animal studies [43] but proceeded with a free-floating device requiring an intact meniscal rim for clinical trials [19].

Interference screws inserted between a graft and bone in transosseous tunnels are well established for fixation of anterior cruciate ligament (ACL) grafts [5, 12, 31], and have also been used for the fixation of a resorbable meniscus replacement scaffold [20, 25, 26].

The aim of this study was to characterise the interference screw fixation system developed for a novel fibre-matrix-reinforced synthetic total meniscus replacement in an ovine cadaveric model. It was hypothesized that the interference screw fixation would provide equivalent mechanics to native meniscus attachments in response to ultimate tensile load and dynamic load.

## Materials and methods

Textile straps with a rectangular cross-section, composed of Dyneema Purity^®^ fibres (DSM Biomedical, Geleen, NL) and identical in material and structure to the textile attachments of the novel total meniscus replacement, were manufactured for this study. Tensile and cyclic creep testing on textile straps determined the peak failure load, peak strain, and creep strain. Fixation testing evaluated slippage following cyclic loading and ultimate failure load of the straps when fixed in ovine tibiae with polyether-ether-ketone (PEEK) or titanium alloy interference screw. Research Ethics Committee approval was not required for this study.

### Tensile testing

Textile straps (*n* = 15) were tested for peak failure load using a screw-driven materials testing machine (5565, Instron Ltd, High Wycombe, UK; tensile failure load accuracy ± 4 N, position accuracy ± 0.02 mm). The straps had a cross-section of 4.4 ± 0.1 mm by 1.5 ± 0.1 mm and a length of 300 mm. The ends of the strap were wrapped around 10 mm-diameter pins and gripped between Instron crosshead clamps with 100 ± 5 mm gauge length as described by ASTM-D5035 [3]. Markings were made across the strap at the inner edge of both clamp jaws to detect any movement during testing, indicating slippage of the strap at the jaws. The straps were loaded in tension at a rate of 100 mm/min until failure [13, 36, 40]. During preliminary testing, it was noted that failure occurred gradually over multiple load peaks as different strands in the fibre straps broke with increasing elongation. Given this, the peak failure load and peak elongation were specified as the maximum recorded load of the first peak, indicating the primary point of failure. The peak strain (%) was determined from the peak elongation and gauge length of each individual strap. Structural stiffness (N/mm) was calculated as the slope of the linear region of the force–elongation curves using linear regression [36, 40].

### Creep testing

Textile straps (*n* = 5) were tested for creep using a servohydraulic materials testing machine (8874, Instron Ltd, High Wycombe, UK). The straps had a cross-section of 4.5 ± 0.1 mm by 1.6 ± 0.1 mm and a length of 300 mm. The ends of the straps were wrapped around 10 mm-diameter pins and gripped between Instron crosshead clamps with 90 ± 4 mm gauge length [3]. Markings were made across the strap at the clamp jaws to detect any movement during testing, indicating slippage of the strap at the jaws. Following 20 preconditioning cycles from 0 to 50 N, the load was increased to 145 N and 1000 cycles between 70 and 220 N were applied at 1 Hz. This has been used previously to represent the loads experienced by the ACL during normal walking [5, 9, 12, 17, 22, 31]. Creep strain was determined at the maximum and minimum load points from the elongation data and gauge length of each individual strap.

### Fixation testing

The interference screw fixation of textile straps in anterior and posterior transosseous tunnels was tested. The anterior and posterior transosseous tunnels replicated the preferred surgical procedure for in vivo ovine studies. At the anterior tunnel, PEEK (BIOSURE PK, Smith & Nephew, UK) and titanium alloy Ti6Al4Va interference screw (QUICK-START, Innovate Orthopaedics, UK) were tested. At the posterior tunnel, single titanium interference screw fixation showed excessive slippage during early cyclic testing, so double fixation was used. Posterior tunnel fixation was implemented using solely titanium interference screw given PEEK screws could not be inserted successfully without blunting.

**Fig. 1:**
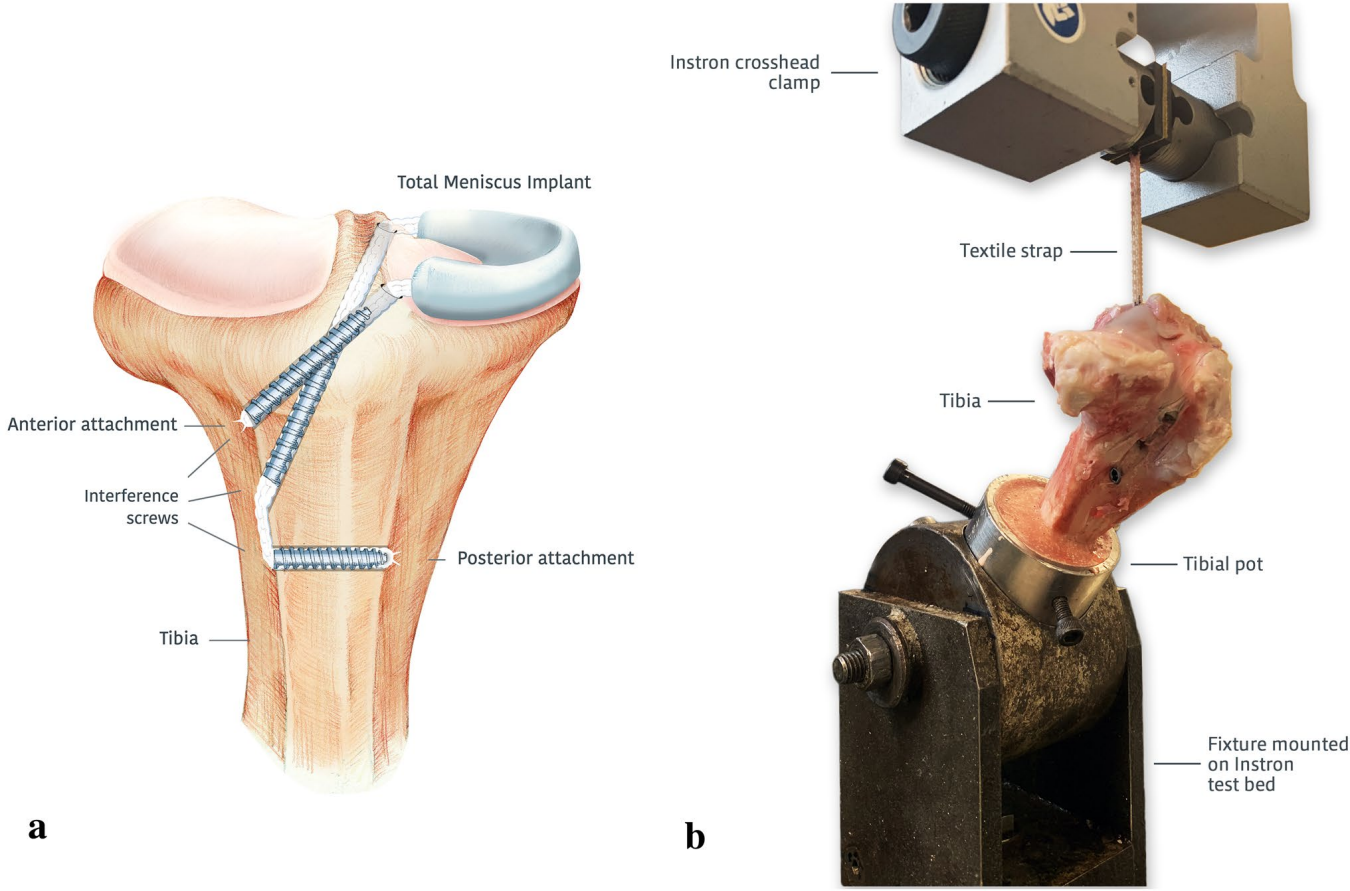
Interference screw fixation of a total ovine medial meniscus replacement. **a** Anterior–posterior view with representation of the ovine medial meniscus implant fixation using interference screws within tibial bone tunnels. **b** Fixation testing setup for evaluating textile strap extension and pull-out load at the bone/interference screw interface in anterior and posterior tunnels. Each bone tunnel was aligned to the tensile loading axis

Fresh-frozen tibias from North of England Mule ewes aged > 2 years and weighing 56–68 kg, with all soft tissues removed, were defrosted immediately prior to testing and kept moist throughout the test. Each tibia was fixed in a stainless-steel pot using three screws and then secured with polymethyl methacrylate (PMMA). In each tibia, an anterior and two posterior 4.5 mm tunnels were prepared using a drill guide. The 20 mm-long anterior tunnel was drilled from the antero-lateral (AL) aspect of the tibial metaphysis up to the plateau at the anterior root attachment of the medial meniscus. The 40 mm-long primary posterior tunnel was drilled from the AL aspect of the tibia, further distal to the anterior tunnel, up to the posterior root attachment of the medial meniscus. A second posterior tunnel was drilled transversely across the distal tibia from the AL aspect to the antero-medial (AM) side to provide double fixation at the posterior attachment, and was 15–20 mm long depending on tibial shaft size (Fig. 1a).

A textile strap was secured at the distal end of each anterior tunnel with a 6 × 20 mm titanium (*n* = 6) or PEEK interference screw (*n* = 5). For the posterior tunnel, the strap was passed through the primary posterior tunnel and then through the secondary tunnel, exiting on the AM aspect of the tibia. A 6 × 25 mm titanium screw was inserted into the distal end of the primary posterior tunnel and a 6 × 20 mm or 6 × 25 mm titanium screw, depending on the tunnel length, was inserted into the secondary posterior tunnel (*n* = 6). The interference screw in the second posterior tunnel provided bicortical fixation. All interference screws were inserted from the AL aspect of the tibia.

The tibial pot was inserted into a fixture that was mounted on the test bed of a servohydraulic materials testing machine (model 8874, Instron Ltd, High Wycombe, UK; cyclic creep load accuracy ± 1.1 N, position accuracy ± 0.2 mm) (Fig. 1b). The fixture allowed the tibial tunnel being tested to be aligned to the loading axis [5, 12]. For the posterior double tunnel, the primary posterior tunnel was aligned to the loading axis. Markings were made across the strap at the clamp jaws to detect any movement during testing, indicating slippage of the strap at the jaws. Following 20 preconditioning cycles from 0 to 50 N, the load was increased to 145 N and 1000 cycles between 70 and 220 N were applied at 1 Hz [5, 9, 12, 17, 22, 31]. The maximum extension at each load cycle was recorded, indicating the combined textile strap creep and slippage from the bone/interference screw interface. A pull-out test was then applied to the strap at 1000 mm/min [12], and the maximum force was recorded.

### Statistical analysis

A power analysis using G*Power v3.1.9.7 [10], based on similar work on the strength of interference screw fixation [5], found that a sample size of six specimens per group would enable identification of significant differences of 75 N with 95% probability and 80% power.

A one-way ANOVA and Tukey’s multiple comparisons test was performed to detect significant differences in pull-out load and extension between each tested fixation method (PEEK/titanium screw and anterior/posterior tunnel) (Prism Version 8.4.3 for Windows, GraphPad Software, La Jolla California USA). Differences were considered significant at *p* < 0.05.

## Results

### Tensile testing

The peak load of the textile straps was 888 ± 137 N (mean ± standard deviation, *n* = 15), elongation 6.2 ± 1.4 mm, strain 6.2 ± 1.3%, and stiffness (slope of the linear region of the force–elongation curve, *R*^2^ = 0.997) was 242 ± 33 N/mm. No slippage of the textile straps was detected at the clamp jaws during testing.

### Creep testing

**Fig. 2:**
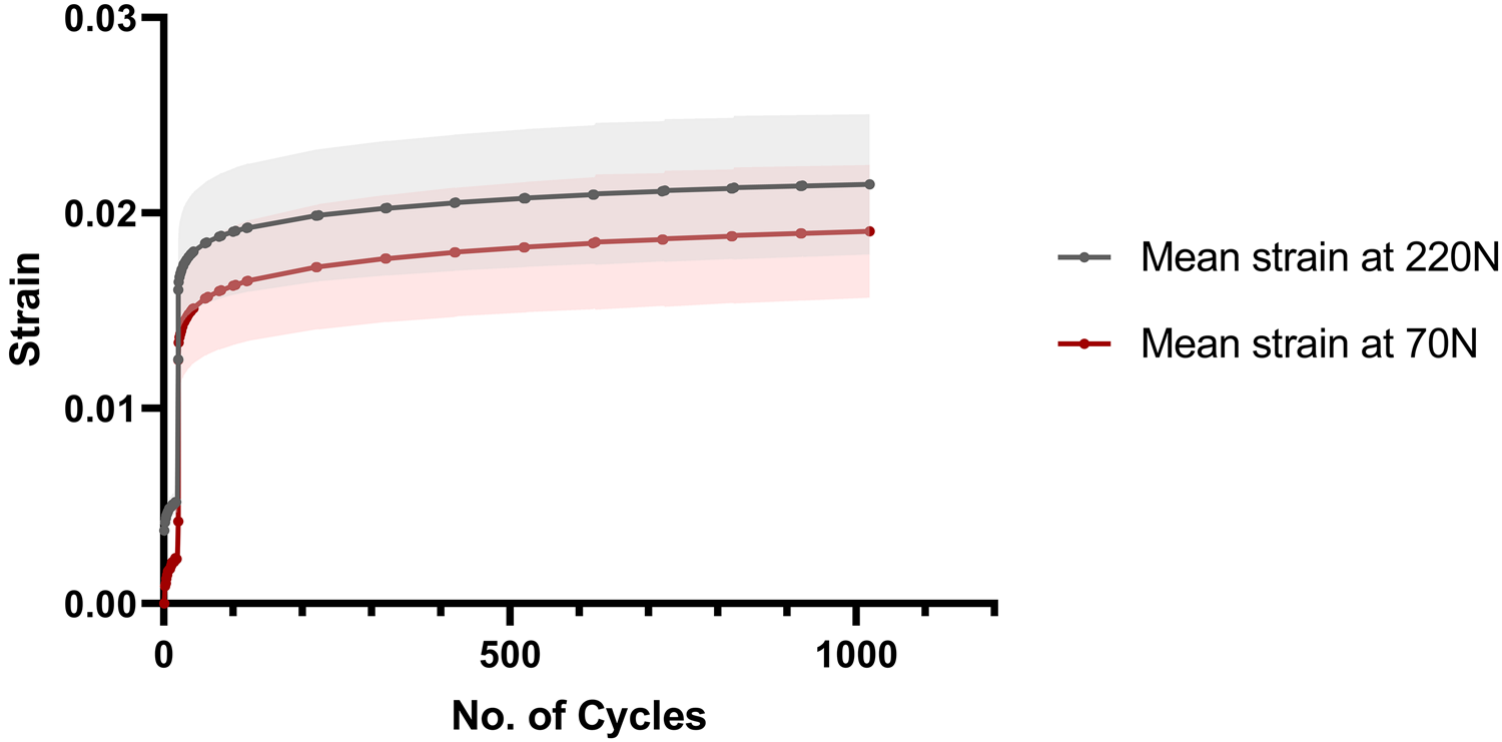
Cyclic strain of textile straps loaded between 70 and 220 N for 1000 cycles. Mean strap strain ± standard deviation against number of cycles (*n* = 5). Average strain and standard deviation at the higher bound of the cyclic load (220 N) is shown in grey; lower bound (70 N) is shown in red

At the first 70–220 N load cycle, the tensile strain of the textile straps at 220 N was 0.016 ± 0.003 (mean ± standard deviation, *n* = 5); this increased to 0.021 ± 0.004 after 1000 cycles (Fig. 2). Therefore, the mean creep strain in the straps was 0.005 after 1000 loading cycles. No slippage of the textile straps was detected at the clamp jaws during testing.

### Fixation testing

**Fig. 3:**
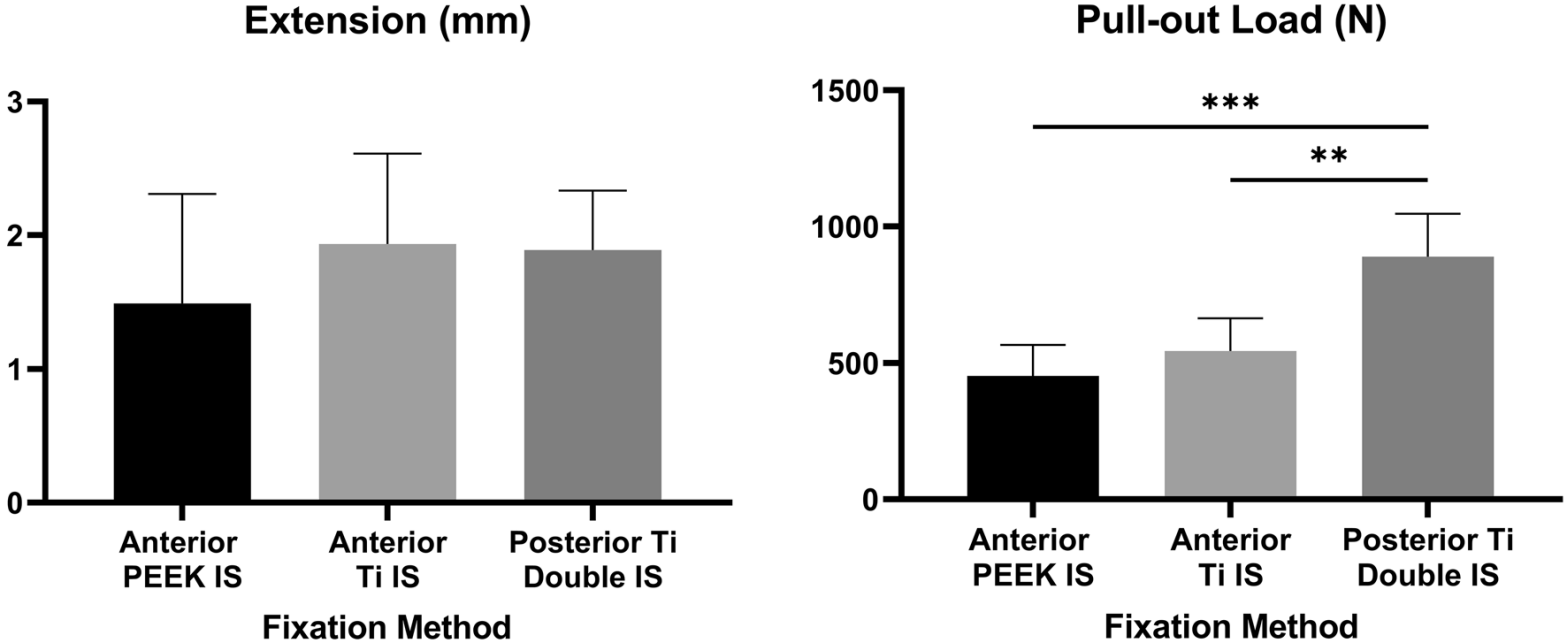
Interference screw (IS) fixation extension (left) and pull-out load (right). Mean + standard deviation reported for both screw types at the anterior and posterior double tunnels (*n* ≥ 5). Adjusted *p* values (one-way ANOVA) represent differences between groups (** significant at *p* < 0.01, *** significant at *p* < 0.001)

**Fig. 4:**
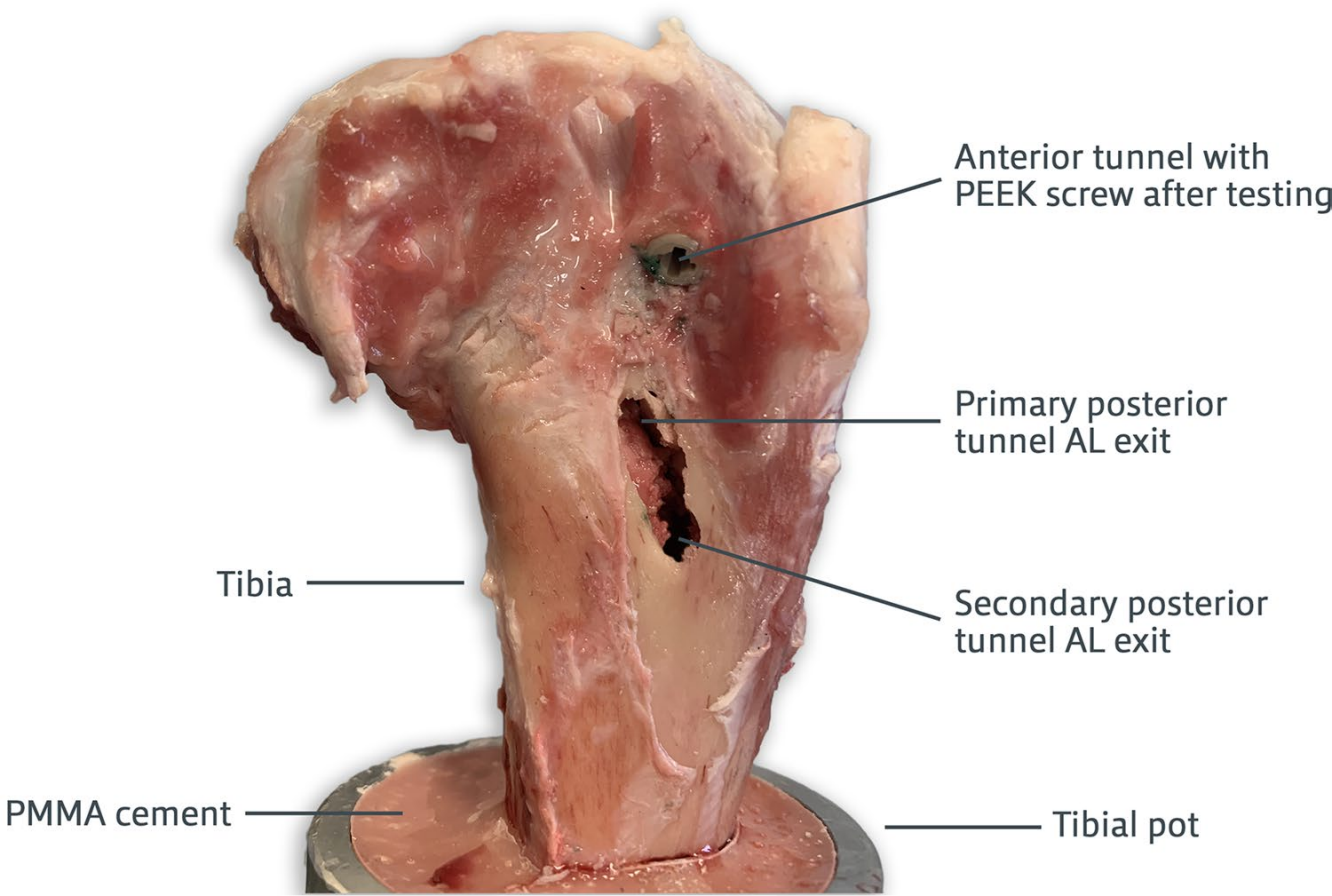
A representative ovine tibia showing bone fracture between the two posterior antero-lateral (AL) tunnel apertures, the main mode of failure of pull-out testing of a textile strap fixed with two titanium interference screws in a posterior double tunnel

The extension (mm) and pull-out load (N) for each interference screw fixation type tested are shown in Fig. 3. No textile straps slipped from the clamp jaws or ruptured during testing. During the pull-out test, the main mode of failure in the anterior tunnel was slippage of the textile strap at the fixation interface until it was completely pulled out of the bone. In the posterior double tunnel, pull-out mainly resulted in the fracture of the tibial diaphysis between the two posterior AL tunnel apertures (Fig. 4). Under visual inspection, straps pulled out from the posterior double tunnel were noticeably damaged by the titanium interference screw threads. ANOVA and post hoc analysis detected no significant differences in extension between the three fixation methods (n.s.). The posterior double-tunnel fixation pull-out load was significantly higher than both the PEEK and titanium anterior tunnel fixation methods (*p* < 0.01), with the two anterior tunnel fixations being comparable (n.s.).

## Discussion

The most important findings of this study were that the fixation strength of the novel total meniscus replacement interference screw fixation system was similar to that of human meniscal root attachments, and that the resistance to slipping under cyclic loads was similar to that of well-established human ACL graft fixation methods, confirming the original hypotheses despite conducting tests in smaller ovine bones. The tensile strength of the double interference screw fixation system was such that during pull-out, fixation failure occurred by tibial bone fracture. These findings support the evaluation of ovine meniscus implants with interference screw fixation in vivo.

Previous studies have characterised the ultimate failure load and stiffness of human meniscus roots [6, 13]. Patel et al. recorded ultimate tensile load for native human meniscus roots and a resorbable meniscus scaffold attachment with antegrade screw fixation in the anterior tunnel and retrograde fixation in the posterior tunnels [24]. The peak strap failure load and the fixation pull-out load in the present study were comparable to human meniscal root strength reported by Patel et al. (735 N anterior and 549 N posterior), Hauch et al. (501 N), and Ellman et al. (583 N) [6, 13, 24].

Based on the biomechanics of the natural meniscus, meniscus replacement attachments require a high stiffness to maintain low strains at peak loads, avoiding implant extrusion, and a high tensile strength to prevent root rupture. Due to differences among specimens, it was not possible to compare the stiffness of the textile attachment strap to previous studies [24]. When scaled by sample gauge length, the strap stiffness was at least 12 times that recorded for human meniscal roots [6, 13]. The peak strain of the textile strap was comparable to the lower end of the strain range reported for human meniscus attachments [13]. These data imply a low risk of rupture or meniscus implant loosening when subjected to physiological loading.

Given that minimal cyclic creep strain was exhibited by the textile strap, it follows that the extension measured during fixation testing was primarily due to slippage at the interference screw fixation interface following 1000 cycles. Clinically, excessive slippage could hinder implant performance in relation to distributing the contact pressures of the knee and providing chondroprotection. Slippage results from this study were within the acceptable range for ACL graft reconstruction in studies with similar loading conditions and fixation systems [2, 5, 9, 11, 17, 31, 39].

Interference screw fixation was chosen for this meniscus replacement implant given its high success rate in ACL reconstruction. Additionally, such a fixation system can be used in arthroscopic procedures and is a well-established procedure among orthopaedic surgeons. For a medial meniscus implant, both the anterior and primary posterior tunnel external apertures were located on the antero-lateral aspect of the tibia to minimise disruption to musculature and innervation to mimic in vivo practice, while still maintaining comparable insertion angles to native meniscus roots. Following a pilot study, double fixation was included on the posterior tunnel to reduce slippage to a value comparable to that of the anterior tunnel, so the pull-out load of the posterior double fixation was at least 63% greater than in the anterior tunnel. The interference screw providing double fixation in the posterior tunnel was inserted from the antero-lateral aspect of the tibia, rather than the antero-medial aspect, to avoid introducing additional incisions on the tibia and also to ensure that the interference screw was embedded in tissue, reducing the possibility of infection during in vivo studies. Furthermore, the fixation testing setup was such that the loading direction of the Instron machine was co-axial with the transosseous tunnel being tested, representing the worst-case scenario. The data suggest that surgical techniques similar to ligament reconstruction may be used for the fixation of meniscal prostheses potentially allowing for faster clinical adoption—the interference screws securing textile straps within the ovine tibial bone tunnels gave similar fixation performance to data published for human ACL reconstructions.

This study has limitations; first, tests were performed on ovine cadaveric tibias and at time point zero, which fail to simulate in vivo conditions, including biological healing and tissue regeneration following the surgical procedure. The possibility of ingrowth into the textile attachments of the novel meniscus replacement implant could further increase the fixation strength and minimise slippage over time. Second, only titanium interference screws were investigated in the posterior tunnel, given that PEEK screws were damaged during insertion at the posterior tunnel. Finally, bone density, which could influence interference screw fixation strength, was not investigated as cadaveric tibias tested were from the same sheep breed and weight range.

## Conclusions

This study found that the novel textile attachment-interference screw fixation system has equivalent ultimate failure load and slippage to native meniscus roots and well-established ACL graft fixation systems, respectively, confirming the hypothesis. These clinically relevant findings support progression to novel fibre-matrix-reinforced total meniscus implant testing in ovine stifles in vivo and will also provide a baseline for future development of human meniscus replacements, in relation to attachment design and fixation methods.

## Abbreviations

ACL: Anterior cruciate ligament
AL: Antero-lateral
AM: Antero-medial
IS: Interference screw
PEEK: Polyether-ether-ketone
PMMA: Polymethyl methacrylate
